# From Past to Present: Transformation of Food Safety Management and Food Safety Culture in the California Almond Industry

**DOI:** 10.1111/1541-4337.70427

**Published:** 2026-02-28

**Authors:** Han Chen, Linda J. Harris, Tim Birmingham, Guangwei Huang, Yaohua Feng

**Affiliations:** ^1^ Department of Food Science Purdue University West Lafayette Indiana USA; ^2^ Department of Food Science and Technology University of California Davis California USA; ^3^ The Almond Board of California Modesto California USA

**Keywords:** almonds, food safety culture, industry collaboration, low‐moisture foods, training

## Abstract

The California almond industry underwent a remarkable transformation in food safety management and culture following outbreaks of salmonellosis associated with the consumption of raw almonds in 2000–2001 and 2003–2004. However, limited studies have examined these changes from a longitudinal perspective. This study documents the transformation of food safety management in the California almond industry over an 18‐year period, explores indicators of change in food safety culture, identifies the key factors driving these changes, and examines the determinants of industry‐wide technology adoption. A multifaceted approach was used, consisting of document analysis and semi‐structured interviews. This study provides a detailed review of the almond industry's responses to the outbreaks, highlighting the industry commodity board's proactive leadership in crisis management, collaborative research efforts, risk assessment, and the development of a mandatory *Salmonella*‐control program to mitigate the risks associated with raw almonds. These measures significantly strengthened food safety management systems across the industry. The industry has also shown a shift in mentality toward food safety over time, evidenced by increased prioritization of food safety, stronger management commitment, and reduced resistance to change. A conceptual framework integrating institutional theory and diffusion of innovation theory is proposed to illustrate how external and internal institutional pressures, along with intervention characteristics, influenced the almond industry's adoption of *Salmonella‐*control interventions. The study offers valuable lessons on proactive, industry‐driven food safety improvements and self‐regulation in enhancing food safety outcomes.

## 1 Introduction

California is the world's leading almond‐growing region, producing 76% of the global almond supply and nearly 100% of the supply in the United States (Almond Board of California [ABC] 2024; California Department of Food and Agriculture 2023). Ranking as the top‐valued agricultural export commodity in California, the almond industry plays a critical role in California's economy (USDA National Agricultural Statistics Service 2023). Almonds are a rich source of monounsaturated fats, protein, dietary fiber, vitamins, and minerals, providing numerous health benefits (Hussain et al. 2021; Kalita et al. 2018; Lee‐Bravatti et al. 2019). With the global demand for almonds as a health food, the industry has a significant responsibility to ensure food safety to protect public health and maintain the industry's reputation.

A food safety management system is essential for minimizing food safety hazards and preventing foodborne illnesses. Two outbreaks of salmonellosis linked to the consumption of raw California almonds occurred in 2000–2001 and 2003–2004, affecting over 200 individuals in Canada and the United States (Centers for Disease Control and Prevention 2004; Isaacs et al. 2005). These were the first documented almond‐associated outbreaks worldwide. A subsequent *Salmonella* outbreak in Sweden in 2005–2006 also implicated raw almonds from California as the suspected source (Ledet‐Müller et al. 2007). These outbreaks revealed gaps in the existing food safety management system in the California almond industry and prompted it to reevaluate its approach to managing food safety. In response, the California almond industry proactively sought to understand food safety issues, improve food safety practices, and implement new standards for food safety control. This resulted in a significant transformation of food safety management in the California almond industry.

Food safety culture, defined as the “shared values, beliefs, and norms that affect mindset and behavior toward food safety in, across, and throughout an organization” by Global Food Safety Initiative (2018), provides a broader organizational framework within which the food safety management system operates. It highlights the interplay between human factors and food safety management systems (de Boeck et al. 2015) and is recognized as a dynamic behavioral phenomenon influencing food safety outcomes (da Cunha et al. 2025). All people in an organization, from management to frontline employees, play an important role in ensuring effective food safety management systems through decision‐making, implementation, monitoring, and verification (Griffith and Motarjemi 2023). Triggers, such as food safety incidents, regulatory changes, or new standards, drive changes in both food safety management systems and food safety culture (Manning et al. 2019). Food safety management systems and food safety culture are intertwined. Food safety culture evolves as management systems develop because the compliance and integration of new practices, standards, and requirements necessitate a deeper understanding of risks and a continued commitment to food safety. Few studies have explored these changes from a longitudinal perspective (Caccamo et al. 2018; Nouaimeh et al. 2018; Spagnoli et al. 2024; Zanin, Stedefeldt, da Silva, et al. 2021).

The ABC, operating as a federal marketing order issued by the U.S. Department of Agriculture (USDA), binds the California almond industry in addressing compliance issues, market development, and research (ABC n.d.‐b). It has a board of directors with 10 members elected from over 7600 almond growers and 99 almond handlers (processors). Board‐appointed committees are filled with over 150 active volunteer members from the almond industry (ABC n.d.‐b). The board plays a vital role in ensuring the safety and quality of California almond production.

Almonds develop on trees as kernels enclosed within a protective shell and outer hull. As the crop matures, the hulls split and the almonds begin to dry. During harvest, mechanical shakers dislodge the nuts, which further dry on the orchard floor before collection. The crop is then delivered to huller and sheller facilities for removal of hulls and typically shells, after which almonds are transferred to processors (handlers) for cleaning, sorting, grading, sizing, and other processing steps (e.g., blanching, roasting, propylene oxide [PPO] treatment) before storage and distribution (ABC 2023).

In 2007, as one of the major outcomes of the outbreak response, the almond industry established a USDA‐mandated *Salmonella*‐control program aimed at reducing the risks of *Salmonella* contamination in raw almonds (Agricultural Marketing Service 2007). This program required an industry‐wide adoption of interventions that achieve a minimum 4‐log reduction of *Salmonella* for all almonds that are grown in California and sold in North America (Canada, Mexico, and the United States). Processors are required to treat almonds with validated process controls if they are not selling to the export market or to direct verifiable (DV) users that have their own validated process controls. Process controls that achieve a ≥ 4‐log reduction of *Salmonella* in almonds are commonly referred to within the industry as “treated” or “pasteurized.” The term “raw” is broadly applied to both almonds that have not undergone a pathogen‐reduction treatment and to those that have, provided they remain unroasted.

The adoption of treatment intervention is a complex process influenced by numerous factors, such as the characteristics of the interventions and environmental pressures surrounding the industry and the individual adopters. In the present study, a conceptual framework that integrated the diffusion of innovation (DOI) theory (Rogers 1962) and the institutional theory (DiMaggio and Powell 1983) was used to understand the almond industry's decision‐making factors in intervention adoption.

The DOI theory, developed by Rogers (1962), is a classical diffusion theory that describes the process of how innovation is communicated and diffused over time among the members of a social system, resulting in the adoption of technology. The rate of innovation adoption is influenced by the characteristics of innovation, including (1) relative advantage: the potential adopters' perceived benefits of innovation over the existing methods; (2) compatibility: the perceived alignment with potential adopters' current values, experiences, and needs; (3) complexity: the perceived difficulties in understanding and using the innovation; (4) trialability: the ability to test the innovation on a limited basis before adoption; and (5) observability: the visibility of the innovation's outcomes. Potential adopters are often presented with an intervention cluster encompassing a set of closely interrelated complementary or alternative interventions, thereby giving them more options to support their decision‐making and encouraging faster adoption of interventions (Dearing 2009; Rogers 1962).

Some researchers have argued that classical DOI theory, while providing valuable insights into understanding technology adoption among individual adopters, may not sufficiently explain the complexities of technology adoption in a broader context that drives institutional change (Bui 2015; Dearing 2008; Redmond 2003). In the context of the food industry, external factors, such as industry trends, government regulations, and consumer demands, can all influence the technology adoption in the industry (Priyadarshini et al. 2019).

The institutional theory is widely recognized and used in organizational research to understand the adoption and diffusion of changes in formal structures, such as policy and practices, among organizations in the same field (David et al. 2019). Organizations surrounded by the same environmental conditions tend to increase their compatibility with the environment to secure legitimacy and thus become similar over time (DiMaggio and Powell 1983). Three types of external pressures, as described by DiMaggio and Powell (1983), influence institutional changes such as industry‐wide technology adoption, including coercive pressure, mimetic pressure, and normative pressure. Coercive pressure refers to external forces exerted on organizations by more powerful entities, such as government authorities or other stakeholders on which the organization depends. Mimetic pressure refers to the pressure that organizations face to imitate others that they perceive to be more successful or legitimate as a response to uncertainty appearing in the organization's environment. Normative pressure, driven by professionalization, arises from norms, values, and standards established and reinforced by professionals through formal education, professional networks, and trade associations (DiMaggio and Powell 1983). Normative pressure also stems from external stakeholders, such as suppliers, customers, and other social entities that shape organizations' practices to conform to the established standards and expectations (Latif et al. 2020).

The California almond industry serves as a case study to illustrate how a commodity sector transformed its food safety management systems and to explore indicators of change in food safety culture in response to the 2000–2001 and 2003–2004 outbreaks. The review synthesizes the factors that shaped this transformation and examines the industry‐wide drivers of adoption of *Salmonella*‐control technologies from 2001 to 2019.

## 2 Methods

The present study used a combination of document analysis and semi‐structured interviews to provide a comprehensive understanding of the evolution of food safety management and culture in the almond industry. Document analysis is a systematic approach for reviewing various types of documents, such as public records, meeting minutes, and newspapers, which are often created without any researcher intervention, and can offer valuable historical insights, track changes and developments over time (Bowen 2009; Morgan 2022). Semi‐structured interviews, on the other hand, are a widely used qualitative research method that allows researchers to gain insights into an individual's experiences, opinions, and emotions relevant to the research objectives and helps uncover the underlying reasons behind observed patterns (Busetto et al. 2020). This combined approach allows for cross‐validation and reduces potential biases that might arise from relying on a single research method (Bowen 2009). The approach entailed (1) a review of available meeting minutes and action plan updates from ABC, and environmental investigation reports of the two outbreaks published by the California Department of Health Services (CDHS); (2) an analysis of news articles; (3) a series of semi‐structured interviews with food safety professionals in the almond industry during the early 2000s; and (4) a series of semi‐structured interviews with current food safety managers from almond processors. The research protocol (IRB‐2023‐1081) was reviewed and approved by the Institutional Review Board (IRB) at Purdue University.

### 2.1 Study 1: Review of ABC Meeting Minutes, Action Plan Updates, and CDHS Environmental Investigation Reports

To track food safety‐related discussions and actions over time within ABC, a review was conducted of the available meeting minutes and action plan updates from ABC's Almond Quality and Food Safety and Services (AQFSS) committee (formerly known as the “Food Quality and Safety Committee”), which was established in 2001 (ABC 2022). In June 2004, ABC unanimously approved a voluntary action plan to ensure that all raw almonds were appropriately treated to reduce *Salmonella* before entering commerce (Agricultural Marketing Service 2007). To keep stakeholders informed about the progress of the action plan, ABC issued periodic action plan updates between 2004 and 2007.

A total of 99 committee meeting minutes, from 2002 to 2019, and 35 action plan updates, from 2004 to 2007, were obtained from ABC (Table S2). The data analysis followed the READ approach suggested by Dalglish et al. (2020), which consists of reading materials, extracting data, analyzing data, and distilling findings. All the meeting minutes and action plan updates were read to become familiar with the material content. Data related to ABC's actions in three key areas were extracted: (1) addressing the microbial concerns for almonds; (2) developing, implementing, and sustaining a mandatory *Salmonella*‐control program; and (3) covering other microbial food safety‐related topics, such as recalls or outbreaks with other commodities. These three key areas served as broad analytical domains for extracting data relevant to the study objectives, rather than representing the themes and categories derived from qualitative analysis. The extracted data, along with relevant details such as the year, date, and number of attendees, were compiled into a Microsoft Word document (Microsoft, Redmond, WA, USA).

To analyze the transformation of food safety management systems, the extracted data were reviewed and inductively organized into themes and categories, with patterns emerging from the raw data. To examine the industry‐wide drivers influencing the adoption of *Salmonella*‐control interventions, the extracted data were deductively mapped onto the pre‐defined themes and categories derived from the conceptual framework that integrated DOI theory (Rogers 1962) and the institutional theory (DiMaggio and Powell 1983). A timeline was then created to illustrate key events and actions in the industry.

In addition to documents from ABC, redacted environmental investigation reports for each outbreak published by CDHS were reviewed to capture government recommendations to the almond industry in response to the outbreaks (CDHS 2002, 2004).

### 2.2 Study 2: News Article Analysis

A systematic analysis of news articles was conducted to provide a broader context. The goals of analyzing news articles were to (1) assess the media coverage of the 2001 and 2004 *Salmonella* outbreaks in almonds and the subsequent mandatory *Salmonella*‐control program; and (2) understand the real‐time public discussion surrounding these events. News articles were accessed through ProQuest (https://www.proquest.com/), which has a comprehensive collection of news from a wide variety of sources, including regional, national, and international newspapers (ProQuest 2024). Previous studies have used the ProQuest database to gain access to newspapers for various research purposes, such as understanding factors affecting news framing (Bolsen 2011), analyzing citations of journal articles in newspapers (Kousha and Thelwall 2019), and exploring newspaper coverage of emerging issues on the topic of interest (Cheng and Edwards 2019).

**FIGURE 1:**
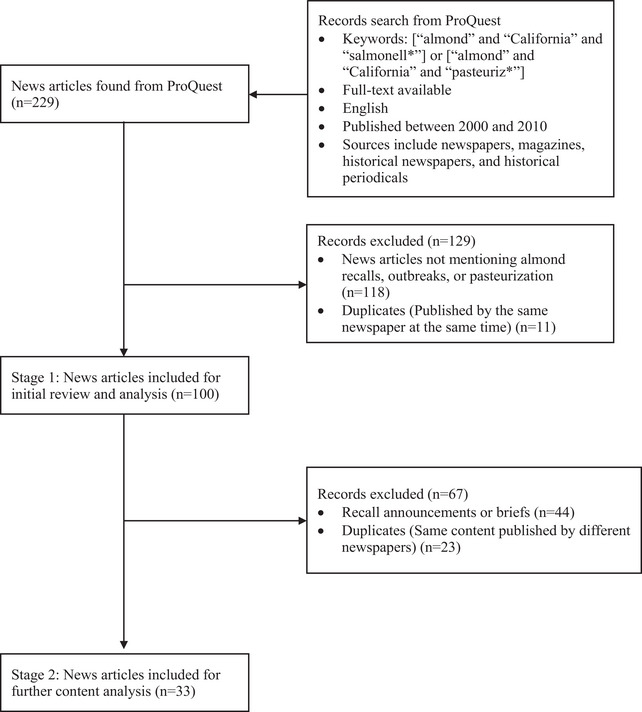
News article search methodology.

A systematic search outlined in Figure 1 was conducted on the ProQuest database through Purdue University Libraries on May 29, 2024. To obtain the news articles related to the two North American almond‐associated *Salmonella* outbreaks and the mandatory *Salmonella*‐control program, the search keywords used were [“almond” and “California” and “salmonell*”] or [“almond” and “California” and “pasteuriz*”]. The additional search criteria were: (1) full text available, (2) published in English, (3) published between 2000 and 2010, and (4) published sources including newspapers, magazines, historical newspapers, and historical periodicals. The search time frame was selected because relevant news coverage was concentrated during this period, whereas meeting minutes were reviewed from 2002 to 2019 to trace discussions and actions over time.

The initial ProQuest search yielded 229 news articles, all of which were reviewed for further analysis. Stage 1 analysis aimed to assess the media coverage of the almond recalls and outbreaks, and the mandatory *Salmonella*‐control program. News articles that did not mention almond recalls, outbreaks, or the mandatory *Salmonella*‐control program, or duplicate copies of the same newspaper issue, were excluded from the Stage 1 analysis. However, articles with duplicate content published by different newspapers were included at this stage to assess the extent of media coverage. These duplicate articles often were the result of wire services, such as the Associated Press, which provided news content to multiple newspapers (Yates et al. 2015). A total of 100 news articles were downloaded and included for Stage 1 analysis. Stage 2 analysis focused on evaluating the public discussion of the events. Recall announcements or briefs that provided information—for example, product withdrawals and safety precautions without additional commentary from experts from universities, government agencies, and industry—were excluded. In addition, duplicate news articles published by different newspapers were excluded at this stage, with only one copy retained for analysis. Thirty‐three news articles were included for Stage 2 analysis.

A coding system was developed in Microsoft Excel (Microsoft), adapted from previous studies (Swinehart et al. 2024; Low and Feng 2024; Archila‐Godínez et al. 2024). In Stage 1 analysis, news article information coded included the news article title, publication year and date, newspaper name, news article type (recall announcement/news brief, or full article), and news event category (2001 recall and outbreak, 2004 recall and outbreak, and mandatory *Salmonella*‐control program). Descriptive data analysis was conducted to quantify the number of news articles that were published on each event.

The coding system for Stage 2 analysis included six major categories: (1) news article characteristics (title, authors, publication year and date, event category, and description); (2) news context (whether almonds were the main topic or mentioned as an example within another discussion); (3) sources of information mentioned in the news and any quotes from these sources; (4) tone and sentiment of the news article (positive, negative, or neutral), with justification for the assigned sentiment; (5) event impacts and responses; and (6) decision‐making factors for the adoption of *Salmonella*‐control interventions (coercive pressures, mimetic pressures, and normative pressures). Two researchers independently coded all the news articles and met to compare and discuss any discrepancies to reach a consensus. Further qualitative data analysis, similar to Study 1, was conducted to categorize the coded data into themes and categories.

### 2.3 Study 3: Semi‐Structured Interviews With Experienced Individuals Involved in Food Safety in the Almond Industry in the Early 2000s

A series of semi‐structured interview questions were developed with the objective of understanding the evolution of food safety management in the almond industry (Table S1). The interview questions were organized into three major sections: (1) history—past experiences with food safety in the almond industry, including perceived food safety awareness and practices, reactions to the almond‐associated *Salmonella* outbreaks, and challenges faced by the industry at the time; (2) present and future—the perceived current state of food safety management in the almond industry and emerging food safety challenges; and (3) the perceived evolution of food safety culture in the almond industry. The questions were reviewed by four food safety experts, including three experts who were involved in the almond industry in the early 2000s, and one expert with extensive experience in qualitative study design. All four experts are authors of this study, and they were not included in the actual interview. The interview questions were finalized based on their feedback.

Fourteen individuals from the almond industry, ABC, regulatory agencies, and consulting and laboratory service companies who were actively involved in food safety in the California almond industry during the early 2000s were identified. A recruitment email was sent to these individuals, followed by multiple reminder emails for those who did not respond. A total of 11 individuals agreed to participate in the study. Interviews were conducted between April and July 2024. Most interviews were conducted via the video conferencing platform Zoom (Zoom Video Communication Inc., CA, USA), with a few conducted by phone at the participants' request. All interviews were recorded with the participant's permission and lasted 30–70 min. Participants were offered $50 in compensation for their time and contribution, although two declined the incentive.

All interview recordings were transcribed verbatim and checked for accuracy by trained researchers (Stoll and Feng 2025; Stoll et al. 2025). The transcripts were then imported into Nvivo 14 (Lumivero, Denver, CO, USA) for qualitative data analysis. The coding process involved two trained researchers: a coder and a checker. The coder developed an initial coding system, following an iterative process in which the codes were continuously revised and refined as all transcripts were coded. The codes were developed both deductively, based on the study objectives and interview questions, and inductively, allowing themes to emerge from the raw data (Saldaña 2021). To reduce bias, the checker independently coded two transcripts and compared the codes with the coder's codes. Any discrepancies were resolved through discussions between the coder and the checker. The coder then refined the codes in Nvivo, and the checker reviewed them. The codes were grouped into related categories and themes, with a consensus reached within the coding team on the final themes, categories, and codes.

### 2.4 Study 4: Semi‐Structured Interviews With Current Food Safety Managers From Almond Processors

Semi‐structured interviews were conducted with current food safety managers from the almond processors to explore the current food safety landscape in the almond industry and assess the impact of previous efforts. A set of interview questions was developed to gain insights into the perceived changes in food safety culture and the factors influencing these changes within almond processors. The interview questions were reviewed by three food safety experts, two of whom specialize in almond food safety; their assessments guided refinements in the final version of the questions. The full interview questions can be found in Chen et al. (Forthcoming). The present study reports a subset of the interview results, focusing on food safety culture.

Participants were recruited both in person and online. Researchers presented the recruitment information and distributed recruitment flyers at the Almond Conference (Sacramento, CA, USA) in December 2023. The recruitment information was also announced at ABC's AQFSS committee meetings and was sent directly to almond processors through the email contacts of the collaborating ABC personnel. The flyers contained a link and corresponding QR code leading to a sign‐up form on the Qualtrics (Provo, UT, USA) website for those interested in participating in the interviews. The sign‐up survey collected: (1) participants' demographic information, including age, gender, education level, and years of experience; (2) company characteristics, including the number of employees and activities involved in the supply chain; and (3) their preferred interview time and contact information. Processing facility personnel who were eligible for participation in the interviews included food safety managers or supervisors managing other employees at almond processing facilities, including hullers and shellers.

A total of 15 interviews were conducted between November 2023 and April 2024. Interviews were conducted and recorded with participants' permission via the Zoom video conferencing platform. The interviews lasted 30–50 min. All interview participants were compensated with $50 for their time and contribution. The qualitative data analysis followed the same approach as in Study 3.

The use of semi‐structured interviews has limitations even with careful study design. The sample size is small, and findings may not be generalized to the entire almond industry. The approach captures subjective perspectives and, therefore, cannot provide sufficient evidence for concluding the level of food safety culture in the almond industry. However, the goal of the present study is not to determine food safety culture level; rather, it is to explore the indicators of change in food safety culture and management based on individuals' experiences and to identify the factors perceived to influence those changes. Using the interview approach allows for the emergence of a broad range of perspectives, captures emotions and contextual insights, and provides an in‐depth understanding of the topic (Mack et al. 2005).

## 3 Results

### 3.1 Characteristics of Data Sources and Interview Participants

In Study 1, 99 ABC AQFSS Committee meeting minutes, 35 action plan updates, and 2 CDPH outbreak investigation reports were reviewed. Between 2002 and 2019, the number of available meeting minutes ranged from 2 to 10 each year (Table S2). The committee met most frequently in 2007 (10 times) and 2008 (8 times). The majority (83%) of the action plan updates were issued in 2004 and 2005 (Table S2). The CDHS outbreak investigation reports covered the period from March 2001 to February 2002 for the first outbreak, and from May to August 2004 for the second outbreak.

In Study 2 Stage 1, 100 news articles were analyzed (Table 1). The content most frequently covered the 2004 almond recalls and outbreak (38%), the mandatory *Salmonella*‐control program (35%), and the 2001 almond recalls and outbreak (27%). Of the outbreak and recall‐related news articles, most were recall announcements or news briefs. In the Stage 2 analysis, 33 news articles were reviewed, including 22 articles featuring the mandatory *Salmonella*‐control program (67%, data is not presented in the table); 7 articles (21%) covered the 2004 almond recalls and outbreak; and 4 articles (12%) covered the 2001 almond recalls and outbreak.

**TABLE 1:** Media coverage of almond outbreaks, recalls, and mandatory *Salmonella‐*control program from Study 2 Stage 1 analysis (*n* = 100).

Topics	Recall announcements or briefs % (*n*)	Full news articles % (*n*)	No. of total news article mentioned % (*n*)
2001 outbreak and recall	20 (20)	7 (7)	27 (27)
2004 outbreak and recall	20 (20)	18 (18)	38 (38)
Mandatory *Salmonella*‐control program	4 (4)	31 (31)	35 (35)

In Study 3, 11 experienced individuals participated in semi‐structured interviews; none were authors of the present manuscript. The participants comprised three from the almond industry (including growers and processors), three from ABC, with one member from the technical expert review panel (TERP), four from consulting and laboratory service companies (including process authorities), and one from a regulatory agency. Most participants were male (73%, *n* = 8). The majority (73%, *n* = 8) began working with almonds in the 1990s; two became involved during the *Salmonella* outbreaks in 2001 and 2004; and one joined the industry in 2007.

In Study 4, a total of 15 current food safety managers from almond processors of different sizes (from less than 20 employees to 500 or more employees) participated in the semi‐structured interviews. Most interview participants were female (60%, *n* = 9), and most participants had a bachelor's or post‐bachelor's degree (80%, *n* = 12). More than half (54%, *n* = 8) of the participants had 5–9 years of experience working in the almond industry, and 33% (*n* = 5) had 10 or more years of experience.

### 3.2 Before 2001: The Uncharted Land

The food safety management systems in the U.S. food industry have a long evolving history, beginning with food safety reforms in the meat industry prompted by Upton Sinclair's *The Jungle*, published in 1906 (Mclntyre 2008), and continuing with hazard analysis critical control point (HACCP)‐based food safety management systems introduced in the 1950s (Manning et al. 2019). However, the microbial food safety landscape (biological hazards) for almonds remained largely unexplored before 2001 (King et al. 1970; Kokal 1969; Kokal and Thorpe 1969; Schade and King 1977; Wehner and Rabie 1970). The Study 3 participants indicated that there was a basic level of awareness regarding general food safety issues, and some discussion around aflatoxins (a chemical hazard), but very limited awareness of the microbial food safety risks specifically related to almonds. A participant said, “The industry hadn't had many experiences with outbreaks or anything of that kind. We were seeing much of what was happening to other nut industries and monitoring those discussions. So I would say there was an awareness, but we weren't as directly involved” (S3P2).

Certain basic food safety practices were already implemented, such as wearing hairnets, cleaning, and sanitation, to meet federal regulations. As a participant explained: “Back then, it was run much like fresh produce packinghouses. You were there to do basic cleaning. You didn't really worry so much about pathogens or any kind of food safety‐related stuff, other than cleaning the equipment on a weekly basis. We were not aware of any potential risks with our product, being a dry product that is shelled out and has a protective hull around the shell. So, prior to then, it was a very simple operation. We had a QA [Quality Assurance] department, but it was really [there] to meet the USDA [U.S. Department of Agriculture] requirements and not so focused on the food safety and the microbiological side” (S3P3).

At the time, the primary focus was on food quality, with microbial food safety as a lower priority. A participant mentioned: “At that point in time, it was a quality mentality, not a food safety mentality. Generally, there wasn't the mindset that you could eat almonds and get sick” (S3P9). The knowledge of microbial food safety risks and controls in the almond industry was described as “uncharted land” by one participant, who noted that “the lessons that were out there from the meat or milk industry really didn't pertain in the same way to almonds” (S3P2).

### 3.3 The 2001 Outbreak: The First Sign of Risk

The first major indication of the potential for microbial food safety risks in almonds arose with an international salmonellosis outbreak attributed to *Salmonella* Enteritidis phage type (PT) 30, which caused 168 confirmed cases of illness, including 157 in Canada and 11 in the United States. In December 2000, an increased number of *Salmonella* Enteritidis infections was detected in Ontario, Canada. Subsequent laboratory tests involving phage typing and pulsed‐field gel electrophoresis (PFGE) helped identify cases of *Salmonella* Enteritidis PT 30 infection (Isaacs et al. 2005; CDHS 2002). Epidemiological investigations ultimately (April 2001) identified raw almonds as the food source of the outbreak (Isaacs et al. 2005). The Canadian Food Inspection Agency (CFIA) issued a Health Hazard Alert on April 12, 2001, warning the public not to consume “California Natural Supreme Almonds as they may be contaminated with *Salmonella* bacteria.” CFIA isolated the outbreak strain from almond samples collected from retail stores, central warehouses, and homes of affected individuals and conducted traceback investigations leading to a single supplier of California almonds. Following these findings, the FDA and the CDHS initiated traceback and environmental investigations in the United States to identify the contamination source (Isaacs et al. 2005). The outbreak‐associated strain of *Salmonella* was recovered from environmental swabs at one almond processor, one of the supplying hullers and shellers, and from at least one orchard associated with each of three growers supplying the huller and sheller (Isaacs et al. 2005; CDHS 2002). Products from the affected processor were recalled in both Canada and the United States (May 7, 2001). Two Study 3 participants highlighted the crucial role of the Canadian government in accelerating the tracing of contaminated almonds: “At that time, the Canadian government was phage typing, and so we were able to trace the origin of the outbreak. The U.S. regulators would not have been able to do the same thing. So, from that point of view, that was helpful, because we were able to trace back to understand exactly where the outbreak had occurred in California very quickly” (S3P10).

Study 3 participants expressed a range of emotions when they first heard about the outbreak, including concern, fear, shock, and surprise about the risks and sources of *Salmonella* and industry reputational damage. A few participants, however, indicated that they were not surprised or afraid, as they were already aware of the potential microbiological risks of consuming raw almonds. Many participants commented that views on the outbreak differed within the industry. Some industry members were open‐minded and were willing to act on the issue, while some were in doubt because they had not personally encountered such an issue and perceived it as an isolated event that would not happen to them. For example, one participant said: “The first outbreak, they traced it back to the field, and all of that product went through one huller and sheller and got manufactured by a single processor, and people were kind of like, ‘well, that's their problem’” (S3P5). In addition, another participant noted that not all industry members were aware of the first outbreak: “Unless you were very involved at the food quality and safety level at ABC, which we [referring to their own company], were not, we didn't know what happened or what the results were. We didn't even know that there was this inherent risk of *Salmonella* that came from the field; it wasn't even a thought at the time” (S3P3).

The findings from Study 2 showed that, besides the recall announcements and briefs, news articles covering the 2001 outbreak often framed it within a broader context, alongside other contemporary foodborne illness outbreaks, such as those involving cantaloupe and spinach. These articles emphasized the increased identification of produce‐associated outbreaks around that time due to the emergence of new pathogens and advancements in pathogen detection technologies. The 2001 almond‐associated outbreak was not featured or discussed as its own problem but as an example of the increasing number of produce‐associated outbreaks. A news article also emphasized that the 2001 outbreak investigation findings revealed the presence and persistence of *Salmonella* in soil in the almond orchards, causing researchers to reconsider how foodborne pathogens can be introduced into the food supply and how to control these risks (Weise 2006).

**FIGURE 2:**
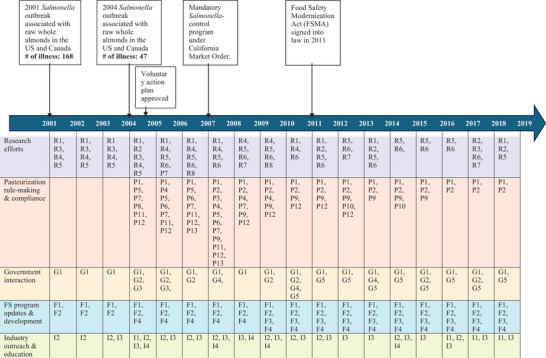
Timeline of key events and food safety‐related actions led by the Almond Board of California from 2000 to 2019. The identifiers (e.g., R1, P1, G1, F1, I1) shown under the timeline correspond to the actions detailed in Figure 3.

**FIGURE 3:**
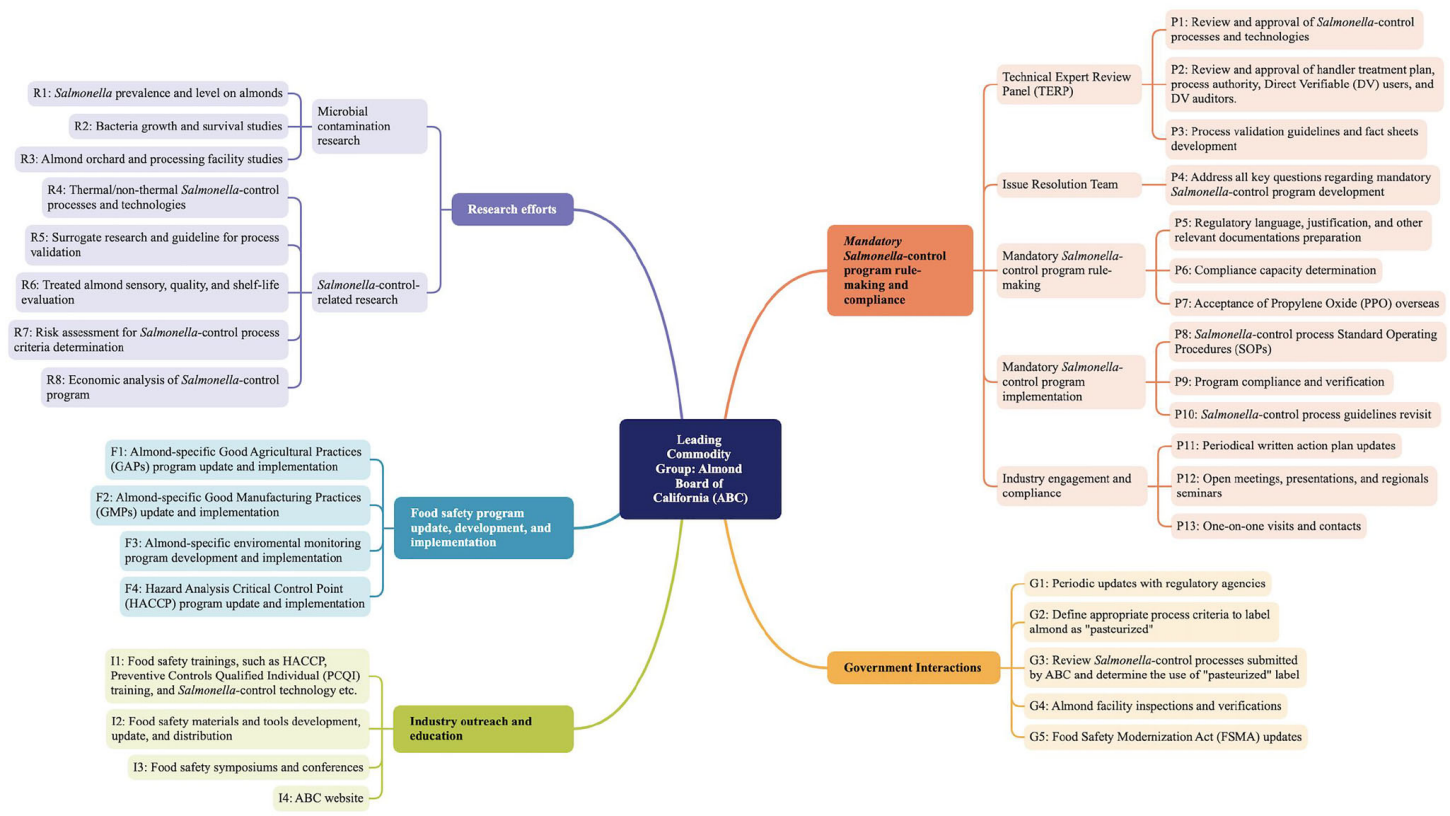
Mind map of the food safety‐related actions led by the Almond Board of California, grouped into five major areas, including research efforts, food safety program development and implementation, industry outreach and education, mandatory *Salmonella‐*control program rule‐making and compliance, and government interactions.

ABC, as a quasi‐governmental organization that administers a federal marketing order for almonds under the supervision of the USDA, was well positioned to take the lead in responding to the outbreak. A participant commented: “The industry kind of looked at the Almond Board and said, ‘fix this’” (S3P9). The timeline of key events and food safety‐related actions led by ABC from 2000 to 2019 is provided in Figure 2, while Figure 3 provides the detailed food safety‐related actions led by ABC, organized into major areas of effort in a mind map.

ABC quickly broadened its focus from promoting almond consumption to addressing food safety issues as the first outbreak occurred. A strike team consisting of eight ABC staff members was formed to handle the outbreak. One participant described that time as: “a 24/7 all hands on deck” (S3P9). The team identified experts who could provide scientific support, had numerous meetings with the regulatory agencies to discuss how to solve the issues, and brought in public relations experts to assist with food recalls. The major challenge for the industry was the limited available information, as this was the first outbreak, thereby making decision‐making particularly difficult. A participant stated: “It was a little bit of a scary time because you're still collecting data, and you don't necessarily have the information that you need to make all the decisions you finally need to make” (S3P4). The industry was looking for information to understand why the outbreak happened and how to minimize the risks of recurrence. One participant said: “Microbial food safety of low‐moisture foods at that time was not a priority. The Almond Board had done some work in the area, but not in a comprehensive and integrated way. So, we spent many long evenings trying to piece together the basic information to understand what we did know and what we didn't know” (S3P10).

Following the first outbreak, several research projects, many of which were funded by ABC, were initiated in collaboration with universities and research companies to understand the cause of the outbreak and explore methods to reduce pathogens in almonds. According to the ABC AQFSS committee meeting minutes in Study 1, research was conducted between 2001 and 2004 in two major areas: (1) microbial contamination studies, including determining the prevalence and levels of *Salmonella* in raw almonds, the environmental persistence of *Salmonella* in almond orchards and processing environments, and the survival on almonds during storage; and (2) studies related to *Salmonella* control, including thermal process lethality validation, PPO fumigation validation, identification of appropriate surrogates for process validation, and exploration of alternative treatment processes and technologies. In addition, some equipment manufacturers became involved following the outbreak, evaluating a range of equipment for *Salmonella*‐control treatments. One Study 3 participant explained ABC's collaboration with the equipment manufacturers: “We spent a fair amount of time working with equipment companies in terms of trying to understand what may and may not work, and the scientific principles involved, whether it's dry heat, whether it's steam, or whether it's a chemical. We tried to stay very much on the generic side of the fence, and then the companies, working with some of the industry members themselves, actually did more work” (S3P10). As a result of the outbreak, one major almond processor began treating its almonds to control *Salmonella*.

In February of 2002, CDHS published its final report on the environmental investigation of the outbreak (CDHS 2002). Recommendations to growers, hullers and shellers, processors, and ABC were included in the report. Growers were advised to implement good agricultural practices (GAPs), establish a recall program, and avoid using uncomposted manure or sewage effluent. Hullers and shellers and processors were advised to adopt the ABC food quality and safety program, follow good manufacturing practices (GMPs), implement cleaning and disinfection procedures with a verification program in place, provide ongoing food safety training for workers, and implement recall and pest control programs. ABC was encouraged to support research into lethal processes, including thermal processes and PPO treatment, to determine the prevalence of *Salmonella* in almonds and the source and geographic distribution of the contamination. They were also encouraged to continue education and training efforts related to food safety.

Ongoing meetings were hosted by ABC with the industry members to update and discuss the latest information, such as the research findings and the interactions with government agencies. Discussions around food safety issues were further expanded into ABC's annual symposium, which had approximately 100 attendees in 2002 (Harris 2002). This 1‐day symposium was first held in 1999 and focused primarily on aflatoxin research. It was redirected to the Food Quality Symposium in 2001. The fourth symposium in 2002 was titled “Food Quality & Safety Symposium” and included research presentations related to the 2001 outbreak and microbiological food safety risks. The symposium was held annually through 2022 (2020 was cancelled due to the pandemic) and remained a key forum for discussing a wide range of food safety issues facing the almond industry (ABC n.d.‐a). In addition, education programs on GAPs, GMPs, and sanitation standard operating procedures (SSOPs) were initiated by ABC. Some food safety materials, such as GAPs newsletters and safe harvest brochures, were developed and distributed to the growers.

### 3.4 The Second Outbreak in 2004: The Confirmed Threat

In 2004, 3 years after the first outbreak, another *Salmonella* Enteritidis outbreak (this time PT 9c) with 47 confirmed illnesses in 12 U.S. states and Canada impacted the California almond industry. In May 2004, a cluster of five related *Salmonella* Enteritidis cases was identified by the Oregon State Public Health Laboratory using two‐enzyme PFGE (CDHS 2004; Centers for Disease Control and Prevention 2004). The similarity in the PFGE patterns of *Salmonella* Enteritidis PT 30 and 9c (Parker et al. 2010) aided the outbreak investigation. In the subsequent interviews, all five patients reported consuming raw almonds within 5 days before symptoms of salmonellosis. The almonds were all from the same brand, supplied by a large California processor, that were sold at chain warehouse stores. The Oregon public health officials associated the outbreak with raw almond consumption and notified the state and federal regulatory agencies and epidemiologists from the United States and Canada (CDHS 2004; Centers for Disease Control and Prevention 2004). More cases were confirmed by identifying *Salmonella* Enteritidis isolates that matched the outbreak PFGE patterns through the national molecular subtyping network (PulseNet), phage typing, and patient interviews.

Trackback and environmental investigations were conducted jointly by the FDA and CDHS. They did not find *Salmonella* in the almond samples obtained from either a patient's home or the affected processor, but they did isolate several serotypes and strains of *Salmonella* from environmental samples collected at the facilities of the affected processor and some of its suppliers (CDHS 2004; Centers for Disease Control and Prevention 2004; Elliot 2005). A Study 3 participant commented that: “For the second outbreak, I don't believe it was sourced from a single handler. It really opened everybody's eyes because this is not that grower's problem, it's not that handler's problem. It's an industry problem” (S3P5). In response to the outbreak, approximately 15 million pounds of raw almonds distributed in the United States and exported internationally were recalled. The chain warehouse stores were able to track customers who purchased the almonds using membership numbers and sent out over 1 million recall announcement letters. There was intense crisis management within the affected processor, and ABC, including handling product recalls, interacting with government agencies, managing public relations, dealing with insurance companies and customers, and working with technical consultants for root cause analysis in the facility. The affected processor began to treat its almonds soon after the outbreak. A Study 3 participant who performed root cause analysis commented: “The company had a very open mind. They wanted to do the right thing. The amount of work that the company did in those 2 to 3 weeks was just amazing. For example, we said the conveyor system was problematic. Two days later, they had changed. They really wanted to put systems together to do the right thing and move forward” (S3P11).

Many Study 3 participants mentioned that the second outbreak confirmed the threat, prompting more industry members to recognize the risks and perceive an urgent need to protect both consumers and the industry. A participant noted: “The second outbreak gives it a more urgent tone because if there are two outbreaks with the same commodity, there is likely to be another until we can put something in place” (S3P6). Some participants also mentioned that they were less surprised when the second outbreak occurred because there was more knowledge about the risks: “By then, we knew that *Salmonella* was pervasive in the growing region, we knew it was in the soil, we knew that the way that almonds were harvested, hulled, and shelled had the potential to facilitate the growth of *Salmonella*… So when the second one happened, we were much more prepared” (S3P5). A note from the ABC AQFSS meeting minutes in Study 1 stated that the instructors of HACCP classes were “impressed by how receptive the attendees were to the concept.” However, a portion of the industry members were still in doubt: “There were a lot of the smaller handlers and growers that aren't in tune with all this. They really didn't understand that this was for real” (S3P9).

Findings from Study 2 showed that the second outbreak resulted in more media attention compared to the first outbreak. All articles, besides recall announcements and briefs, focused on the description and discussion of the 2004 outbreak, including the number of illnesses, the number of products being recalled, and the affected processor's responses to the outbreak. Some news articles also included interviews with experts from universities, government agencies, representatives from ABC, almond growers and processors, and consumers. Major reactions captured in the news articles were consumer shock over the rare occurrence of *Salmonella* in almonds and the industry's concerns about its public health impact, reputation damage, and economic consequences.

Soon after the outbreak, ABC took an active lead in pushing for industry‐specific food safety programs (Figures 2 and 3). A participant said: “It was a matter of stepping up the work we were doing with other companies, manufacturers, as well as researchers, and government authorities to really start determining what steps and procedures we could put in place to avoid a third outbreak” (S3P2). According to the ABC AQFSS committee meeting minutes in Study 1, many efforts went into food safety program development and implementation, as well as industry outreach and education. Task forces were formed within ABC to further update and develop user‐friendly GAPs and GMPs targeting the almond industry. In ABC's action plans, they conveyed to the industry the importance of complying with the food safety programs: “Even when pasteurization [*Salmonella‐*control program] becomes mandatory, GAPs and GMPs will remain critical components of food safety. The board is finalizing revisions to increase their usability and practicality.” In addition, various food safety education seminars, workshops, symposiums, and conferences were held for industry members.

In June 2004, the ABC board of directors unanimously approved a voluntary action plan to ensure that all raw almonds entering commerce were appropriately treated to reduce the risks of *Salmonella* contamination. The plan aimed for all domestic and export shipments of raw almonds to undergo treatments to achieve a 5‐log reduction in *Salmonella*, except for those shipped directly to manufacturers that would further treat the almonds, referred to as DV users. At the time, compliance with the *Salmonella‐*control program was voluntary, and ABC was looking into making the program mandatory. A Study 3 participant from ABC commented: “We're a federal marketing order. Any procedures that the industry would have to comply with would have to be done through rule‐making” (S3P2). Meanwhile, the industry members were strongly encouraged to participate in the voluntary *Salmonella‐*control program to reduce the risks of another outbreak before the mandatory program took effect.

In the environmental investigation report of the outbreak published by CDHS in 2004, the recommendations to growers, hullers and shellers, and processors remained similar to those in the report of the first outbreak (CDHS 2002, 2004). However, additional recommendations were made for processors, including implementing a pathogen reduction step and creating clean zones to prevent cross‐contamination between almonds before and after treatment.

In October 2004, ABC established a TERP, which consisted of five scientists from different backgrounds, to conduct technical reviews of the efficacy of the *Salmonella*‐control processes and technologies in *Salmonella* reduction, develop standardized protocols for the industry, and provide scientific advice. In addition, ABC maintained ongoing engagement with government agencies, regularly sharing information. A Study 3 participant said: “Both California Health and the FDA were very aware of what we were setting up to try and come up with, [such as] standardized protocols for which surrogate to use and the best procedures for the different steam technologies being used. At the time, PPO was more prominent, so chemical procedures [were developed]. So yeah, a lot of effort went into the development of TERP” (S3P10).

Extensive ABC‐initiated (and often ABC‐funded) research efforts were undertaken in collaboration with universities, research companies, and equipment manufacturers. The various research projects initiated since the first outbreak, such as a survey of the almond crop for the presence and levels of *Salmonella*, evaluation of outbreak‐associated orchards, process validation, and surrogate research, continued. Research reports on blanching, oil roasting, and commercial PPO validation studies were submitted for FDA review and feedback. In September 2004, the FDA determined that the PPO process parameters did achieve a 5‐log reduction of *Salmonella* in almonds (ABC 2008; Danyluk et al. 2005), which became the first FDA‐recognized pasteurization treatment protocol for almonds, meaning that almonds treated under specific PPO treatment parameters in the protocol could be labeled as “pasteurized.” Later, in July 2005, the blanching and oil roasting processes were recognized as 5‐log pasteurization treatments for almonds after additional studies were conducted per the FDA's feedback (Abd et al. 2012; ABC 2007a, 2007b; Du et al. 2010; Harris et al. 2012). Following the letters of determination from the FDA, standard operating procedures (SOPs) for the pasteurization treatments were developed and distributed to the industry (ABC 2007a, 2007b, 2008). Additional studies explored alternative *Salmonella*‐control processes and technologies that included an assessment of the impact of these interventions on almond sensory, quality, and shelf‐life characteristics, a quantitative microbial risk assessment for pasteurization process criteria determination, and an economic analysis of the mandatory *Salmonella*‐control program. According to the Study 1 action plans, by July 2004, 10 other possible *Salmonella*‐control processes and technologies had been investigated, including dry heat processes (e.g., dry roasting), moist heat processes (e.g., steam pasteurization), and non‐thermal treatments such as chemical sanitizers, cold plasma, radio frequency, and ultraviolet (UV) treatments.

In the continued investigation of potential treatment processes and technologies, there was discussion regarding whether it was necessary to achieve a 5‐log reduction of *Salmonella*. Ongoing research has shown that a 5‐log reduction is challenging to achieve for some processes (e.g., dry roasting or steam treatments) without negatively impacting almond quality and sensory characteristics. A Study 3 participant explained: “One of the key things that we found out was that, in the steam processes available at that time, if you tried to get a 5‐log reduction for every sample, you ended up causing the skins to separate from the product, and you materially changed the look of the product. So, in this case, almonds, how many log reductions are really needed to keep it safe, right?” (S3P3). According to the action plan updates in Study 1, the industry needed to provide adequate data to the FDA, demonstrating that an alternative treatment process criterion with less than a 5‐log reduction would be adequate to protect consumers.

An initial quantitative microbial risk assessment of almonds, evaluating the risks of salmonellosis from consuming almonds, was initiated in early 2005 and completed in late 2005 (Danyluk et al. 2006). This study was feasible because data collection funded by ABC, starting in 2001 on the prevalence and levels of *Salmonella* in raw almond kernels (Danyluk et al. 2007) and on the survival of *Salmonella* on stored almond kernels (Uesugi et al. 2006) was available. The risk assessment was discussed at ABC AQFSS meetings as early as February 2005, presented in a poster form at the Institute of Food Technologists annual meeting in July 2005 (Danyluk et al. 2005), and discussed at more than one ABC meeting in the fall of 2005. In the poster and some of the subsequent discussions, the risk assessment outcomes associated with a hypothetical 5‐log process were compared with 3‐ and 4‐log target reductions. The risk assessment manuscript, which included an assessment of a target 5‐log reduction, was submitted for publication in December 2005 and was published in July 2006 (Danyluk et al. 2006).

The risk assessment characterized the predicted public‐health impact of various log‐reduction treatments and provided the scientific basis for ABC's risk management decision to adopt a minimum 4‐log reduction of *Salmonella* as the performance standard for almonds, later incorporated into the mandatory *Salmonella*‐control program (Agricultural Marketing Service 2007; Lapsley and Harris 2022). Subsequent risk assessments conducted in 2012 (Lambertini et al. 2012) and in 2017 (Santillana Farakos et al. 2017) confirmed that a minimum 4‐log reduction treatment substantially reduces the predicted risk of illness.

### 3.5 Navigating the Path to Mandatory *Salmonella*‐Control Program: A Turbulent Time

**FIGURE 4:**
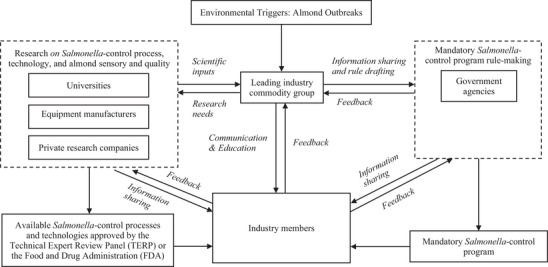
Network of key players and their interactions in the development of the mandatory *Salmonella*‐control program.

The process of moving the *Salmonella*‐control program from voluntary to mandatory was complex (Figures 2 and 3). Figure 4 presents the key players, including ABC, industry members, research entities (university, research companies, and equipment manufacturers), TERP, government agencies, and their collaborative interactions during the mandatory program development through information sharing, feedback exchange, communication and education, and rule‐making.

Extensive discussions took place to “develop a regulatory program that was feasible, enforceable, and financially viable for the industry” (P3S2). One of the main driving forces of the mandatory *Salmonella*‐control program development and implementation was the capacity and availability of treatment processes and technologies that did not compromise almond quality, sensory characteristics, and shelf life. In February 2005, a rule‐making task force was established within ABC to develop regulatory language, justification, and other relevant documentation supporting the mandatory program. In August 2005, an issue resolution team (IRT) composed of members with different expertise from industry, government agencies, and industry associations was established to address key questions and find consensus on specific issues in the mandatory program development.

The initial goal of the mandatory *Salmonella*‐control program was to ensure, by August 1, 2005, that all almonds for both domestic and export shipments were adequately treated. However, the capacity for PPO and the availability of other technologies at the time limited the industry's ability to treat all almonds. In addition, resistance arose from many customers overseas who did not want to receive treated almonds. A Study 3 participant explained: “The majority of what's being exported is going into ingredient usage where it will be pasteurized. Then, do you need to pasteurize that prior to shipment?” (P3S2). To move the program forward, the possibility of having a mandatory *Salmonella*‐control program for domestic shipments was explored. Due to the North American Free Trade Agreement (NAFTA), the mandatory program was extended to cover shipments within North America, including the United States, Canada, and Mexico. A Study 3 participant described it as a notable change: “Some people said, ‘Maybe we should pasteurize the almonds for North America, but not the rest of the world.’ That was a sea change when we had that discussion because 70%–80% of our production went offshore, which meant far fewer almonds would need to be pasteurized. It filtered back to our little company, where we are 90% export. So, all of a sudden, we're not going to own a pasteurizer. We don't need that because we don't do much domestic work in North America” (S3P1).

In the rule‐making process for the mandatory program for North America, there was heavy debate around whether the DV program, originally developed as a short‐term domestic option to address capacity concerns, should be temporary. This program allowed domestic shipments of unpasteurized almonds to manufacturers who had their own validated treatments. The ABC AQFSS committee meeting minutes from 2005 in Study 1 showed that some major snack and confectionery manufacturers who used almonds as ingredients were concerned about the impact of treated almonds on their product quality and shelf life, and the additional associated costs. A Study 3 participant also commented on the resistance from the large manufacturers: “We had pushback from those customers on the pasteurization, because they were like, ‘We're going to put it in our product anyway. We're going to deal with it. We're going to cook it. We don't need pasteurized almonds’” (S3P9). In addition, some perceived that the DV program provided the industry with more time to address the quality and technological issues and also made it easier for small processors to comply with the mandatory program because most of them had a higher percentage of exports than domestic sales. Conversely, some people worried that the industry could not track and verify the safety of the unpasteurized almonds after they were sold.

Following the debate on the DV program, the ABC board of directors decided to remove the sunset clause of the DV program in August 2006 and added the labeling requirements of the term “unpasteurized” on containers of the shipments to the DV users, as well as export shipments outside North America, to the regulatory language to track the unpasteurized almonds and protect the market. A Study 3 participant perceived the DV program as a key to getting many manufacturers to buy into the mandatory program: “I think that the DV program really helped alleviate a lot of food quality concerns that the manufacturers had and really brought them on board as supporters” (S3P5). Another Study 3 participant was emotional about not being able to enforce 100% treatment of domestic and export shipments of almonds: “We had put blood, sweat, and tears into this over the years… We had 4 to 6 years of trying to get this program through since the first outbreak. And then, it kind of got scuttled, which actually made me go home and do a lot of soul searching with [laughs] a bottle of whiskey on the counter as to whether I really wanted to be part of this if they were going to short shrift it. And I decided that, obviously, it's better than not having one at all. So, we stuck it out and continued down that road” (S3P9).

To ensure the inclusion of various industry segments in the rule‐making process, another major consideration was the organic almond and in‐shell almond sectors, which had limited appropriate treatment technologies identified at the time. Organic growers and processors were very concerned that treating almonds would disqualify them from being organic. The industry, however, perceived the importance of holding the organic sector to the same standards as other commercial almonds. A Study 3 participant explained: “The ABC took a firm stand, and we said, ‘everything has to be pasteurized,’ because if an organic almond becomes contaminated with *Salmonella*, and it makes somebody sick, the whole industry is going to be branded with that unhealthy image” (S3P1). To address the needs and concerns, ABC members communicated with the USDA National Organic Program to explore treatments that meet the organic standards. It was later determined that there were treatments available for organic almonds, such as steam processing and traditional industry thermal processes used for roasting and blanching. In terms of in‐shell almonds, PPO treatment was the only option as of March 2007, but as previous validation studies were conducted with almond kernels, additional research was needed to address the questions raised by TERP to fully accept the use of PPO for in‐shell almonds. Later, in 2008, a steam treatment technology (H2O Express) was tested and accepted by TERP for in‐shell almond pasteurization; that became the first alternative treatment to PPO treatment for in‐shell almonds and also met the organic pasteurization requirements.

In November 2006, the proposed mandatory *Salmonella*‐control program was submitted to the USDA and published in the Federal Register for public comments in December of that year. The final rule was issued under the federal marketing order (7 CFR Part 981) in March 2007. It required, by May 31, 2007, all handlers to submit a handler treatment plan to ABC outlining how almonds, if not shipped directly to DV users or export markets, would be treated to achieve a minimum 4‐log reduction of *Salmonella* before shipment. This plan served as a roadmap describing each handler's approach to treating their almonds to meet the rule and was reviewed and approved by ABC. The mandatory compliance date for the rule, when handlers were required to implement the treatment as outlined in their submitted plan, was set as September 1, 2007.

**FIGURE 5:**
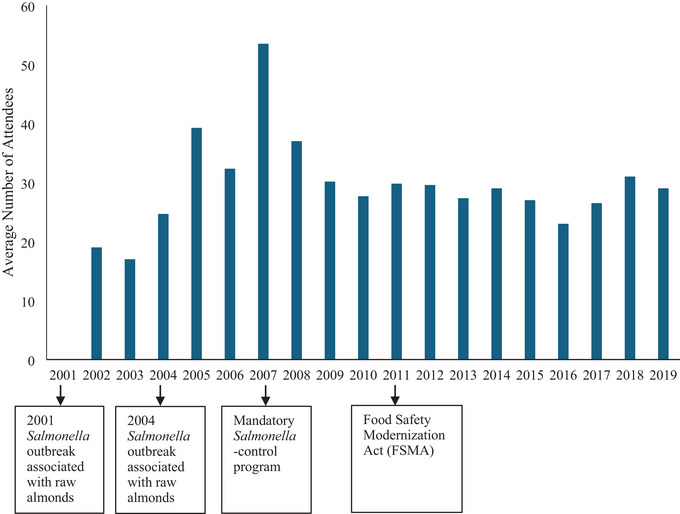
Trends in meeting attendance by year based on meeting minutes (2002–2019).

The annual mean number of attendees of the AQFSS committee meetings from 2002 to 2019, as recorded in the meeting minutes, is shown in Figure 5. There were fluctuations in meeting attendance over the 18‐year period, with a peak in 2007, in which the average number of attendees reached more than 50 per meeting. After 2007, attendance generally decreased and remained at an average of around 30 attendees per meeting, which was overall higher than the average number from 2002 to 2003.

Findings from Study 2 showed that the mandatory *Salmonella‐*control program (pasteurization) had been a topic in news articles (*n* = 22) since 2005, with the most articles published in 2007, the year the rule was implemented. Most news articles (68%, data not shown in table) were written in a neutral tone without favoring or opposing the program, while 18% were in a negative tone emphasizing the concerns of *Salmonella*‐control interventions, and 14% were in a positive tone emphasizing the benefits of *Salmonella*‐control interventions. Over half of the news articles (55%) were conflict‐framed, presenting both positive and negative views toward the mandatory *Salmonella*‐control program among different groups. Supporters indicated that the program could protect consumers while maintaining the quality, taste, texture, and appearance of almonds. A few news articles focusing on other outbreaks and recalls, including an *Escherichia coli* outbreak associated with spinach in 2006 (Centers for Disease Control and Prevention 2006) and a pistachio recall in 2009 (U.S. Food and Drug Administration 2009), praised the proactive actions taken by the almond industry after the two outbreaks and showcased the use of *Salmonella*‐control interventions as a way to control microbial risks. However, critics, particularly organic almond producers and raw food enthusiasts, argued that treated almonds could no longer be considered truly raw to meet the consumers' demands for raw almonds. Some claimed that this led to the loss of the organic raw food businesses and asserted that the “raw” label on treated almonds could be misleading to consumers. In addition, there were concerns about the financial and regulatory burdens that the program exerted on producers and about the health risks of using chemical treatment PPO. According to the ABC AQFSS committee meeting minutes in Study 1, ABC had been addressing some of the concerns highlighted in the news articles, including investigating the reduction of PPO residue after treatment, which supported PPO as a safe substance for almond pasteurization. Efforts also included obtaining acceptance of PPO use for almond exports to other countries and gathering information regarding the definitions of “raw.” It was later clarified that treatments, including PPO, steam, or moist heat, were considered minimal processing of almonds, allowing treated almonds to still be considered “raw.”

Industry readiness was key to the successful implementation of the mandatory program. Thus, in 2007, ABC's efforts were focused on two main aspects, according to the ABC AQFSS meeting minutes in Study 1 (Figures 2 and 3). The first aspect was to assess and ensure sufficient treatment capacity for the industry. ABC was working with TERP in reviewing more *Salmonella*‐control technologies and processes, approving DV users and auditors who would audit DV users, and approving process authorities who could validate treatment processes in the facilities. Validation guidelines on several processes had been developed and distributed to process authorities. In addition, some treatment processes, such as the traditional thermal processes, were already in place and just needed to be validated as a pasteurization process. A Study 3 participant explained: “The industry as a whole had some of the pasteurization methods in their facilities. They just didn't realize it. A lot of the industry had oil roast applications, dry roast applications, and blanching applications. They just had not proceeded to validate that equipment…. It's not that you have to go out and buy all new equipment to make this. Also, many handlers and growers had propylene oxide chambers” (S3P7). As of August 2007, the capacity of validated processes was determined to exceed the treatment demands.

The second aspect was getting all industry members on board and prepared for program compliance. A Study 3 participant said: “I think the challenge with any industry group is getting widespread buy‐in and commitment to do something different…. It just requires a little more effort, extra time, extra information, and extra data” (S3P6). Another participant echoed: “We had to convince all the rest of the people in the industry that we had to do this. So, the onus was [on] the leadership at the Almond Board” (S3P1). According to the ABC AQFSS committee meeting minutes and action plan updates in Study 1, extensive industry outreach and engagement efforts including open meetings, presentations, regional seminars, an annual Food Safety & Quality symposium, and periodic written action plan updates had been undertaken since the approval of the voluntary action plan to keep the industry apprised of the mandatory program development and implementation and to combat misinformation. A Study 3 participant highlighted: “Throughout this time period, it was just ongoing communications to all of the stakeholders. This became a priority for a lot of what we were doing; it was also the catalyst for a lot of our food safety programs” (S3P2). In addition, ABC initiated one‐on‐one visits with almond handlers in 2007. A Study 3 participant explained: “We created a PowerPoint presentation, and we divvied up the entire list of handlers—it's about 100 handlers. Each handler got a personal visit from an executive member at the Almond Board who walked them through the presentation, answered their questions, and explained to them why the board believed that we needed to do something proactively” (S3P5).

There were two influential activities highlighted by participants in terms of getting industry buy‐in to the mandatory *Salmonella*‐control program. Some participants indicated that a public meeting involving a well‐established epidemiologist and regulators from government agencies played an important role in helping the industry, especially small growers and handlers, understand the inherent risks of agricultural products grown in open fields and pushed the industry toward taking action. Commenting on this meeting, one participant confided: “As I was going back to the parking garage, getting my car, and driving home, he scared me. But I think he woke us all up. We needed to have that awakening. At the time, I wasn't grateful. But when I look back now, I'm grateful for his comments… He said, ‘If you don't fix this problem, we will fix it for you, and you likely won't like it’” (S3P1). Another effort highlighted was a straight talk video produced by ABC in 2007, featuring the affected processors of the first outbreak who shared their experiences and lessons learned from the outbreak, which was perceived to be very effective in communicating the risks to the industry. A Study 3 participant emphasized: “I think that [the video] was what convinced a number of the people in the industry that this is a problem we all have to face now because we're on FDA's radar screen, especially after the second outbreak” (S3P10).

### 3.6 Post‐2007: After the Implementation of the Mandatory *Salmonella*‐Control Program

Various efforts led by ABC continued in 2007 and beyond (Figures 2 and 3). One major focus was verifying compliance with the mandatory program. Soon after the program went into effect in 2007, ABC took the lead on conducting compliance audits and verifications to ensure that handlers were adhering to their treatment plans, which had been approved by ABC. Concurrently, audits of DV users were also conducted. According to the ABC AQFSS meeting minutes in Study 1, the handler treatment plan verification form was further revised in 2009 with an emphasis on basic GMP requirements and almond production flow for identifying potential post‐process contamination issues. The revision aimed to create a more robust audit program.

ABC continued to revisit and update the food safety programs, including GAPs, GMPs, and HACCP. In 2009, ABC collaborated with external consultants in developing a pathogen environmental monitoring program for the almond industry. Relevant guidance documents and tools for the food safety programs were developed and distributed to facilitate program implementation. In 2012, 5 years after the mandatory *Salmonella*‐control program was implemented, there was a push for revising and enhancing the pasteurization process validation guidelines to be more user‐friendly. Ongoing surrogate research also informed further refinements to the *Enterococcus* guidelines, which were updated in 2014 (ABC 2014).

Beyond surrogate research, the ABC AQSFF committee funded research activities that included surveys of the almond crop for presence of *Salmonella*, investigations in the 2001 outbreak‐associated orchards, pathogen survival studies in soils and stored almonds, evaluation of approaches to dry sanitation, new treatment processes and technologies development, almond quality, sensory, and shelf‐life studies, and economic analysis of the mandatory program. In addition, ABC maintained ongoing interactions with government agencies. Beyond the mandatory *Salmonella*‐control program, another area of focus in government interactions was obtaining updates on the proposed rules under the Food Safety Modernization Act (FSMA) signed into law in 2011. Both the Produce Safety Rule (PSR) and Preventive Control Rules for Human Foods and Animal Foods had a direct impact on the almond industry. Due to the mandatory *Salmonella*‐control program and other previous efforts in food safety program development, the almond industry was prepared for the new regulations. A Study 3 participant commented: “When FSMA came up, we've got another government regulation, and how's this going to work? But actually, we were already there. We were ahead of the other nut industries because we had the pasteurization step. Even though it was a difficult time, and I would not want to relive it, it set us up to be prepared for food safety as we move forward” (S3P1).

In 2016, the ABC started working with the FDA to explore the options for a possible PSR exemption for almond growers. Later, in 2019, the FDA decided to exercise enforcement discretion in enforcing the FSMA PSR for entities growing, harvesting, packing, or holding almonds due to the implementation of the mandatory *Salmonella*‐control program (U.S. Food and Drug Administration 2019). Nonetheless, growers were still expected to understand the rules and follow proper food safety practices. A Study 3 participant explained: “They have what's called an Enforcement Discretion. So, because of the mandatory program, it is not enforced to the extent that it would be on a fresh produce grower. The Almond Board spent a great deal of time educating folks on it. Most of these people are going to be required to have done it because whoever's handling their crop is going to want them to know that. It's about managing the risk” (S3P4). ABC developed various FSMA decision‐making tools and guidelines to help industry members understand and comply with the regulatory requirements. In addition, ABC hosted several industry‐specific preventive control qualified individual (PCQI) training and grower PSR training sessions to help prepare the industry members for compliance with the regulations. Over the years, numerous conferences, annual food safety symposia, workshops, and webinars were hosted for industry outreach, education, and regulation updates to further support the industry.

### 3.7 The Current Status of Food Safety Management in the Almond Industry

Most Study 3 participants perceived the almond industry as currently having good food safety management systems in place. A participant commented, “Now as an industry, things are under better control than they used to be” (S3P12). However, they also noted the need for continuous improvement with ongoing research and education to stay on top of the new research findings, technologies, and best practices. A participant said: “I think it's very important for the industry and each individual company to stay on top of the latest technologies, research findings, and best practices that regulatory agencies have that would improve our food safety even better. You can't protect what you don't know” (S3P3).

Many Study 3 participants mentioned economics as an emerging food safety challenge. Participants commented that almond producers struggled with the increased production costs and lowered product pricing, which impeded the ability of producers to afford all the tools needed for food safety controls. A participant further explained the concern: “I think the emerging food safety challenge is the economic challenge because it costs money to produce these crops and to do it profitably. The concern I have is that people might look for cheaper alternatives that were not being evaluated safely and could introduce a food safety risk that they hadn't planned for. So, there's got to be a balance… Everything is connected to everything else. You solve one problem, but you might create another” (S3P4).

### 3.8 Reflections on the Evolution of Food Safety Culture and Food Safety Management

When looking back on the long journey since the first outbreak, many Study 3 participants perceived a shift in mentality toward food safety, reflected by a deeper understanding of the microbial risks, heightened food safety awareness, and improved food safety management, which heightened the sophistication of the industry. A participant said: “I remember when the first outbreak happened. The hulling and shelling operations were fairly rough, like just one step up from the farm. Now, when you visit a hulling and shelling operation, it looks like a food manufacturing facility. They're sealed better, they're cleaner, and the way that they're able to handle the product is much more mindful of cross‐contamination” (S3P4). Many participants expressed that they were very proud of the outcomes of the efforts. One participant commented: “This was a pot of controversy, it really was. Those meetings in Modesto were contentious and difficult. I didn't look forward to those meetings, but I'm very proud of how it all turned out” (S3P1). A few participants emphasized the need to keep the food safety mindset and to remain proactive: “A lot of the people that were involved in this initial process, like myself, we're getting towards retirement age. So, hopefully, that awareness of what happens if you don't keep the food safety practices in mind is there and is continued in future generations” (S3P4).

Study 3 participants perceived two factors as most influential in shaping food safety management in the almond industry throughout the years: the two outbreaks and ABC's leadership. Many participants perceived the *Salmonella* outbreaks in almonds as the cornerstone of changes toward food safety. A participant said: “The most significant factor was the fact that they had a few outbreaks, which showed them that they needed to improve their system, and they did” (S3P11). Besides the almond outbreaks, participants indicated that other outbreaks and recalls that happened around the time also increased overall awareness of food safety. One participant explained: “I worked with other commodities as well. In 2009, there was a significant outbreak or recall with pistachios, so it started to impact other crops grown in this area as well, because these people don't just grow a single crop, they grow a myriad of crops. So, they started to realize it's not just them, there are other people facing the same issues” (S3P4). This was also reflected in the ABC AQFSS meeting minutes in Study 1. The accounts of various outbreaks and recalls that were discussed during the meetings included a salmonellosis outbreak associated with dried cereal in 2008 (Russo et al. 2013), peanut butter (Centers for Disease Control and Prevention 2009) and pistachios in 2009 (U.S. Food and Drug Administration 2009), and a listeriosis outbreak associated with cantaloupe in 2011 (Centers for Disease Control and Prevention 2011), which also were lessons for the almond industry.

Many Study 3 participants perceived ABC's leadership as a significant factor. Participants praised ABC for its leadership in engaging and guiding the industry members, fostering collaboration in problem‐solving with different parties, and its willingness to invest in research to develop solutions based on science and facts that accommodate the needs of various industry members. A participant said: “I think the Almond Board does deserve credit for the actions they took, the specific steps they put in place scientifically. There was data to support their actions. Overall, it seemed to me a very balanced and scientific‐based approach to developing effective intervention steps… When you want to change an industry, it does require leadership from within the industry. Regulators can develop regulations, inspect and quarantine contaminated products, and write warning letters, but generally, regulators are slow to change an industry compared with what the industry can do from within” (S3P6). Some participants perceived that the establishment and implementation of the mandatory *Salmonella*‐control program made a big impact on the overall food safety management of the industry. A participant commented: “It was a positive step forward. Given how the industry works, the Almond Board came up with a very credible and workable intervention to minimize the risk of additional outbreaks” (S3P6). As shown in the ABC's AQFSS committee meeting minutes (Figure 5), the average meeting attendance in 2007, when the mandatory *Salmonella*‐control program was implemented, reached the highest across the 18‐year period from 2002 to 2019, highlighting the attention this program received and its significant influence on the industry.

Most Study 3 participants perceived that the food safety culture in the almond industry had improved compared to the early 2000s. A few participants emphasized that the food safety systems improved overall in the industry, but the food safety culture varied by company, with different levels across the industry. Study 4 participants who were current food safety managers for almond processing companies were asked about perceived changes in food safety culture. Sixty percent of the participants had 5 years or more of experience in their current company; most perceived an improvement in the food safety culture in their own companies over the years.

The improvements that participants from Study 3 and Study 4 perceived included: (1) increased food safety priority, reflected in increased budgets for food safety management, more frequent food safety meetings, and the hiring of additional food safety personnel; (2) more management support and commitment to food safety; and (3) reduced resistance and fewer questions regarding changes or actions aimed at enhancing food safety. Participants identified both external and internal factors affecting improvements in food safety culture in almond companies. External factors included food safety regulations, third‐party certifications, customer expectations, and prior outbreaks and recalls. Most participants indicated that the food safety regulations implemented were a significant driving force. One participant explained: “The new regulations, preventive controls that came out, it's not really new anymore. But that kind of raised the bar for everyone” (S4P1). In addition, many participants mentioned that food safety culture had become one of the requirements for third‐party certifications: “I think it's probably in all the GFSI [Global Food Safety Initiative] schemes now. We follow the SQF [Safe Quality Food] code, and it became a requirement 2 years ago to have a food safety culture” (S4P13). Internal factors identified included the company's willingness to invest in food safety, ongoing communication about food safety, and an increased number of resources available for employees.

### 3.9 Reflections on *Salmonella*‐Control Technology Adoption Decision‐Making Through the Lens of Institutional Theory and DOI Theory

**FIGURE 6:**
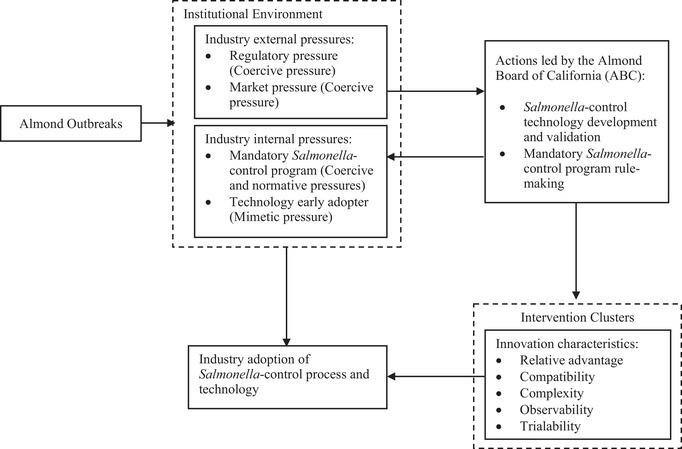
Conceptual framework of decision‐making factors influencing industry adoption of *Salmonella*‐control processes and technologies, adapted from institutional theory (DiMaggio and Powell 1983) and diffusion of innovation theory (Rogers 1962).

Figure 6 presents the conceptual framework of the decision‐making factors influencing the industry adoption of *Salmonella*‐control processes and technologies, adapted from the institutional theory (DiMaggio and Powell 1983) and the DOI theory (Rogers 1962). It illustrates that the adoption decisions of industry members were influenced by institutional pressures, including industry external pressures triggered by the almond outbreaks and industry internal pressures arising from ABC‐led food safety actions, as well as by the characteristics of the developed and validated *Salmonella*‐control processes and technologies resulting from ABC‐led research efforts.

#### 3.9.1 Industry External Pressures

The two *Salmonella* outbreaks associated with almonds resulted in external coercive pressures from both government agencies and the market, including consumers and customers, compelling the industry to take action to resolve problems and shortcomings. Pressures from government agencies intensified following the second outbreak. A Study 3 participant said: “There was an ultimatum from the government during the second outbreak: ‘You are going to fix the problem.’ During the first outbreak, it was: ‘You better be thinking about this because of all the implications.’ But during the second outbreak, it was: ‘No, if you don't do that, we were going to do it’” (S3P1). The two outbreaks emphasized the urgency for the industry to take proactive measures. Another participant noted: “Repeated outbreaks in the same commodity are a strong warning to industry and to public health regulators that there's an underlying problem here that really needs to be fixed” (S3P6).

Market pressures also played a critical role in driving industry actions, particularly after the second outbreak, which affected several major almond customers. A Study 3 participant said: “You don't want to lose the gold star customers” (S3P5). In addition, there were concerns about the impact of the outbreak on the growing almond market at the time. An almond grower, quoted in a news article from Study 2, commented: “It's a real shame because it comes at a time when we are seeing increasing supply, increasing demand, and higher prices” (Hirsch 2004). Both outbreaks, especially the second one, received media attention, raising public awareness about the issues and further amplifying market pressures. A news article highlighted the importance of addressing food safety concerns to avoid losing the market, quoting an expert: “They [the industry] had to do something themselves, or else they're going to lose their market” (Grady 2007).

#### 3.9.2 Industry Internal Pressures

Under external pressures, ABC took the lead in responding to the outbreaks and addressing microbial concerns in almonds. A key outcome of these actions was the development of the mandatory *Salmonella*‐control program, which created both coercive pressures and normative pressures within the industry. Mimetic pressures emerged as early adopters in the industry successfully implemented *Salmonella*‐control processes and technologies.

The adoption of *Salmonella*‐control interventions was influenced by internal coercive pressures and normative pressures arising from the mandatory program. ABC, as a quasi‐governmental organization, institutionalized *Salmonella* control as the industry standard through rule‐making. The mandatory *Salmonella*‐control program was added to the outgoing quality control requirements of the federal almond marketing order under the supervision of the USDA, creating coercive pressures for compliance among industry members. A Study 3 participant commented: “This pasteurization rule applies to everybody and is monitored very closely” (S3P3). Beyond regulatory mandates, the program also created normative pressures, as compliance was viewed as a shared moral obligation within the industry to protect consumers. One Study 3 participant explained: “What I see is the moral obligation that the industry acknowledges that it has to create and sell a healthy and safe product” (S3P5). This norm was reinforced in ABC's action plan updates and meeting minutes, which emphasized that “food quality and safety are the top priority for the almond industry.”

Mimetic pressures further influenced adoption as early implementation of *Salmonella*‐control processes and technologies by some almond processors set examples for the rest of the industry. Several major almond processors began treating their almonds before the program was mandated, as reported in contemporary news coverage. In a context of uncertainty about available technologies and concerns about potential business consequences of delaying action, the legitimacy and demonstrated effectiveness of these early adopters provided credible models that encouraged broader industry uptake. A Study 3 participant also commented: “I think after the second outbreak, a lot of the industry members could see that the industry was going in that direction. They could see that their competitors were buying equipment and that it wasn't going away. So eventually, I think the majority of the industry came on board” (S3P7).

#### 3.9.3 Intervention Characteristics

Industry members' adoption decisions also were influenced by the interventions' characteristics, including relative advantage, compatibility, complexity, observability, and trialability, according to the DOI theory (Rogers 1962). To ensure the industry‐wide adoption of *Salmonella*‐control interventions, ABC initiated and expanded extensive efforts in collaboration with various universities, research companies, equipment manufacturers, and government agencies to develop, validate, and review a range of *Salmonella*‐control interventions that could meet the needs of all industry segments and ensure sufficient treatment capacity. These efforts resulted in a cluster of approved *Salmonella*‐control interventions, such as PPO treatment, steam and moist heat processes, roasting, and blanching, allowing processors to select the options most compatible with their facility needs.

Industry members were familiar with the approved interventions, reducing uncertainties in the adoption process. Study 3 participants noted that many facilities had equipment for oil roasting, dry roasting, blanching, or PPO chambers, and processors were required only to validate the existing equipment and processes rather than to purchase new equipment. One participant explained: “One of the reasons why we forged ahead and were able to do this so quickly is we learned early on that a fumigation method or technique that we used, PPO, was actually resulting in some pasteurization. In fact, when we applied it to certain parameters and with a vacuum, it consistently resulted in pasteurization. So that was a very familiar tool” (S3P5). Another participant added: “The industry had access to these pasteurization methods. Once they understood that they had some of the tools that they needed, then they started using them” (S3P7). The prior experience with the interventions reduced the complexity of adoption and alleviated concerns about compatibility, trialability, and observability, because the equipment was already in place.

ABC was also actively communicating the availability of existing *Salmonella*‐control equipment to the industry, enabling industry members to comply with the mandatory program more easily. ABC hosted various educational seminars, workshops, and the annual Food Quality & Safety Symposia that covered *Salmonella*‐control processes and technologies, which provided opportunities for industry members to explore and compare the relative advantage, compatibility, complexity, observability, and trialability of different technologies. Having such outreach and educational events supported industry members' decision‐making on the technology adoption. The significance of the impact of relative advantage on technology adoption was highlighted by a Study 3 participant, who stated: “They're also an industrial group, meaning, what you do has a cost, this cost has benefit, and the benefit outweighs the cost, this is really important” (S3P11).

## 4 Discussion

This study showcased the significant transformation of food safety management in the almond industry, along with indicators of change in food safety culture, triggered by two *Salmonella* outbreaks in the early 2000s. Unlike animal products such as meats and dairy, which have a long‐known history of microbial risks dating back to the 19th century (Boor et al. 2017; Institute of Medicine [U.S.] Food and Nutrition Board 1990), the almond industry remained largely unaware of the microbial risks of raw almonds before the first outbreak, with few exceptions outside of the United States such as coconut and peanut products (Harris et al. 2024), nuts had not previously been linked to outbreaks of foodborne illness.

The 2001 *Salmonella* outbreak marked a pivotal moment for the almond industry, but at the time, it was not broadly recognized as such. Many industry members perceived the incident as an isolated event attributable to those particular growers and processors and questioned whether broader action was needed (Isaacs et al. 2005). This reaction likely reflected both the industry's limited experience with foodborne pathogens in tree nuts and the influence of optimism bias, a psychological tendency to believe one is less likely than others to experience negative events (Gouveia and Clarke 2001; Sharot 2011).

The second outbreak in 2004 (Centers for Disease Control and Prevention 2004) challenged this view and catalyzed a shift in perception across the industry. In the interim between the outbreaks, research supported by ABC and the USDA had begun to improve understanding of microbial risks. By 2004, 2 years of statewide survey data had shown that *Salmonella* could be recovered from approximately 1% of 100‐g samples of raw almond kernels (Danyluk et al. 2006). *Salmonella* was not recovered from almonds linked to the 2004 outbreak; however, several strains unrelated to the outbreak were isolated from environmental samples collected at the affected processing facility. These findings reinforced the reality that *Salmonella* contamination could occur outside of identified outbreaks and underscored the need for proactive intervention. The response trajectory aligned with the progression pattern described by Lynch et al. (2009) in which an initial outbreak reveals a novel transmission pathway and subsequent events lead to greater acceptance of the risk and the expectation that it may recur.

To protect the almond industry and consumers, ABC, as an agricultural commodity board with responsibilities and resources to maintain almond markets, was ideally situated to take the lead in understanding and addressing the issues that emerged. Such a commodity board is in a favorable position to bring different parties together and develop balanced solutions that meet their needs (Young and Hobbs 2002). Similarly, in the case of a 2009 *Salmonella* outbreak associated with peanut butter (Centers for Disease Control and Prevention 2009), the industry commodity group, the American Peanut Council, took the lead in crisis communication and coordination, which was reported to be effective because the overall peanut butter sales did not decline that year (Irlbeck et al. 2013).

ABC demonstrated leadership in crisis management, reflected in its prompt response and decision‐making, extensive collaboration, and regular communication. Timely responses to the outbreaks are key in addressing public health concerns and maintaining public trust in the commodity (Wilson et al. 2017). A delay in taking action could lead to harsh public criticism, as denial and downplaying are common initial reactions in such crises, which could potentially mislead decision‐making (Motarjemi 2023). There were many unknowns at the time of the first outbreak because the food‐pathogen pairing was novel. Establishment of the link between the food and the pathogen and understanding the routes of contamination can be difficult (Lynch et al. 2009). Limited available information impeded the process of deciding the next steps, according to Study 3 participants. However, waiting for all the facts to be clear before deciding what to do is often inadvisable (D'Auria and De Smet 2020). ABC did not downplay the situation or delay taking action. Shortly after the first outbreak, ABC formed an internal crisis management team, engaged experts with different expertise to investigate the outbreak, and developed and updated food safety programs. Soon after the second outbreak, ABC approved a voluntary action plan for treating all almonds entering the market to protect public health, which was later developed into a mandatory *Salmonella*‐control program. These measures, complemented by the rapid actions taken by the affected processors, such as issuing extensive product recalls and implementing corrective actions, were vital in minimizing industry reputation damage, preventing future incidents, and gaining consumers' trust (Motarjemi 2023).

Collaboration and communication were essential to ABC's crisis management. They quickly established collaboration networks with numerous parties, including universities, regulatory agencies, industry members, research companies, equipment manufacturers, and other relevant entities (Figure 4) to understand the outbreaks and investigate proper *Salmonella*‐control processes and technologies. These collaboration networks allow rapid information sharing and problem‐solving under intense conditions (D'Auria and de Smet 2020; Motarjemi 2023). ABC also established TERP to perform independent reviews of research and pasteurization processes and technologies and develop standardized protocols for the industry. Such an independent scientific panel plays an important role in ensuring research quality and efficacy, and in guiding commodity boards in the proper allocation of research funding (Bowen 2017). Research progress and findings were regularly shared with TERP, industry members, and government agencies to gather feedback and inform future directions, which provided scientific inputs to guide decision‐making.

Collaboration and communication among various parties are not only essential in crisis management but also in ensuring an unbiased decision‐making process (Motarjemi 2023). ABC worked closely with government agencies on the mandatory *Salmonella*‐control program rule‐making process and obtained clarification on compliance with varied regulatory requirements, such as the labeling requirements and organic standards. ABC also formed specialized teams, such as the rule‐making task force and IRT, to respond to specific needs and ensure an efficient rule‐making process. Throughout the process, ABC maintained open and transparent communication with industry members through regular meetings, seminars, periodic written updates, and one‐on‐one conversations. These communication activities kept industry members informed about the rule‐making progress and enabled them to provide feedback to shape a practical and feasible *Salmonella*‐control program, and these communication channels were critically important in getting industry members on board with the program.

Crisis prevention is particularly important for ensuring long‐term food safety and strengthening the industry's reputation. Moving forward after the outbreaks, ABC continued its collaborative efforts with the industry to enhance overall food safety management systems, including verifying compliance with the mandatory *Salmonella*‐control program, updating and implementing food safety programs, advancing relevant research, and providing educational resources. Together, these initiatives enabled the development of a proactive risk management system to prevent future food safety incidents. Wilson et al. (2017) indicated that proactivity, transparency, credibility, consistency, prioritizing consumers, and adhering to established protocols and procedures are critical for building consumer trust in the food system. Having a robust risk management system also ensures confidence in the food safety regulatory process. The effectiveness of these strategies was evident in the almond industry's preparedness during the implementation of the FSMA, as well as its ability to secure enforcement discretion on certain FSMA rules.

After years of effort, most Study 3 participants believed that the almond industry had established a robust food safety management system. However, they also identified economic pressure as an emerging challenge that could hinder continued progress. Rising production costs and declining product prices have reduced profit margins, potentially limiting producers' ability to invest in food safety controls. Recent reporting supports these concerns: almond producers have been struggling with persistently low prices and high operational expenses (James 2024). Prior research similarly identified cost as a major barrier to growers' compliance with food safety practices (Chen et al. 2021).

From a food safety economics perspective, decisions about allocating limited resources to food safety are influenced by factors such as the public health burden of illness, the direct and indirect costs of outbreaks, and consumer attitudes and willingness to pay for safer food (Focker and van der Fels‐Klerx 2020). To address economic constraints, Wu et al. (2008) suggested providing financial incentives, developing cost‐effective control techniques, and offering education and outreach programs to promote the adoption of food safety controls. Among these, education and outreach are particularly important for building a culture of food safety among almond producers and supporting decisions that prioritize prevention even in the face of economic pressure. Ultimately, the long‐term costs of foodborne illness outbreaks and recalls far outweigh the investment required for prevention (Focker and van der Fels‐Klerx 2020; Ribera et al. 2012).

Reflecting on the evolution of food safety management, it is evident that the California almond industry has made significant advancements since the early 2000s. Study 3 participants identified the two outbreaks coupled with ABC's leadership in the response as the two most significant contributing factors that affected food safety management in the almond industry. The two almond outbreaks were the wake‐up calls for changes toward food safety (Manning et al. 2019). Motarjemi (2023) also indicated that crises are usually the origin of improvements in food safety management in industrialized countries. For example, the Jack‐in‐the‐Box outbreak in 1993, associated with 600 illnesses and the deaths of four children from consuming undercooked hamburgers contaminated with *E. coli* O157:H7, prompted rapid movements toward risk‐based food safety management approaches, and ultimately reformed the meat and poultry inspection systems worldwide (Murano et al. 2018). The 2009 *Salmonella* outbreak associated with peanut butter, which caused more than 700 illnesses and 9 deaths, sped up implementation of the reportable food registry (U.S. Food and Drug Administration 2010), underscored the demand for stricter food safety regulations, and provided additional incentive for the passage of the FSMA in 2011 (Guthrie 2009; Leighton 2016).

In the case of salmonellosis outbreaks linked to almonds, ABC played an important role in transforming food safety management by leading the development of almond industry‐wide self‐regulation standards. As one Study 3 participant noted, meaningful change in an industry often requires leadership from within. ABC exemplified this through the development and implementation of a mandatory *Salmonella*‐control program, which is overseen by the USDA. Sharma et al. (2010) noted that successful self‐regulation in the food industry depends on transparent standards, meaningful and clear objectives and pre‐defined benchmarks, accountability, and oversight by an appropriate regulatory body. In addition, the involvement of diverse stakeholders such as industry, academia, and government is critical to the development and sustainability of self‐regulatory standards (Sharman et al. 2020). ABC's approach demonstrates how coordinated industry‐led action can effectively drive improvements in food safety management.

Most Study 3 and Study 4 participants perceived improvements in the food safety culture within the almond industry over the years, noting a shift in mentality that included increased prioritization of food safety, stronger management support and commitment, and reduced resistance to change. The present study identified a combination of external and internal factors that influenced the changes in food safety culture, which aligns with prior research, which also showed that both the company's internal and external environment influenced a company's food safety culture (Nyarugwe et al. 2020; Spagnoli et al. 2023). External factors, including food safety regulations, third‐party certification requirements, and heightened customer expectations, affected the overall food safety environment in the industry. Previous studies have reported that food safety governance, achieved through food safety regulation, public and private standards, and enforcement, was an important factor influencing companies' food safety culture and performance (Kirezieva et al. 2015; Nyarugwe et al. 2020). To sustain business, meet customer expectations, and remain competitive, companies need to comply with food safety regulations and standards, which enhances their overall food safety awareness. In addition, beyond the outbreaks associated with almonds, numerous outbreaks and recalls have been associated with nuts, seeds, and other low‐moisture foods since 2001 (Acuff et al. 2023; Harris et al. 2024; Harris and Yada 2022; Wason et al. 2021). The meeting minutes from ABC AQFSS documented discussions about many of these outbreaks and recalls, which helped remind the industry of the importance of food safety and the need to continuously raise awareness across the sector.

While external factors set the external food safety environment, internal factors drive the establishment of the food safety culture within individual companies. Internal factors identified were the company's willingness to change, ongoing food safety communication, and an increased number of resources, reflecting the commitment of company management personnel to food safety, communication, and education efforts. The commitment of top‐level managers is particularly important because they have the power to allocate resources for food safety management, and they are in an ideal position to drive down food safety messages to the entire company (Chen et al. 2024; Neal et al. 2012). Pai et al. (2024) reported the significant role of leadership and organizational commitment in building a strong food safety culture by influencing employees' food safety performance. Similarly, Taha et al. (2025) found that leadership, whether transactional or transformational, is positively associated with employees' increased commitment to food safety and hygiene practices. In addition, continuous food safety communication and education were essential to engage employees in food safety conversations and decision‐making and enhance their commitment to food safety, which were critical to building a positive food safety culture in the company (Chen et al. 2024; Global Food Safety Initiative 2018; Zanin, Stedefeldt, and Luning 2021). Together, both external and internal factors contributed to a shift in food safety culture in the almond industry.

The present study proposed a conceptual framework of the decision‐making factors influencing the industry adoption of *Salmonella*‐control interventions. This framework considered the impact of the institutional pressures, both external and internal to the industry, and the characteristics of interventions themselves on the potential adopter's decision‐making. Institutional pressures influence the necessity of adopting an intervention, the characteristics of which influence the determination of the specific intervention to adopt. In the case of the almond industry, external coercive pressures from government agencies and market demands following the outbreaks prompted the commodity board to initiate self‐regulation as a response to restore legitimacy, which is important to rebuild public trust and gain support from stakeholders and society (Bruton et al. 2010).

The mandatory program established *Salmonella* control as a regulatory standard for safety and a shared norm for food safety within the almond industry, introducing internal coercive pressures and normative pressures to drive compliance. Meanwhile, early adopters of *Salmonella‐*control interventions created internal mimetic pressures for the rest of the industry to follow suit to avoid potential market losses and maintain competitiveness. DiMaggio and Powell (1983) indicated that companies tend to imitate others they perceive to be legitimate in response to uncertainty. A previous study conducted by Latif et al. (2020) found that companies under higher institutional pressures, including coercive, normative, and mimetic, were more likely to adopt environmental management accounting to address environmental issues. Coercive pressures were reported to have the strongest effect on decision‐making compared to normative pressures and mimetic pressures (Arranz et al. 2022; Latif et al. 2020).

Beyond the institutional pressures, the availability and capacity of *Salmonella*‐control interventions were critical for industry‐wide adoption. The present analysis demonstrated that ABC collaborated with numerous parties to develop and validate a cluster of interventions, offering potential adopters various options and facilitating faster adoption (Dearing 2009; Rogers 1962). According to Rogers (1962), interventions are more likely to be adopted when they demonstrate higher relative advantages, observability, compatibility, trialability, and lower complexity. ABC's collaborative efforts included incentivizing research on *Salmonella*‐control processes and technologies, providing scientific data and findings, and delivering targeted outreach and education programs on *Salmonella‐*control interventions and other food safety controls, which increased the perceived relative advantages, observability, and trialability of interventions while reducing the perceived complexity to support the adoption process. Technology adoption is an iterative process in which ideas develop and evolve through interaction, collaboration, and knowledge and resource sharing (Vargo et al. 2020). Thus, continuous engagement and collaboration are critical for driving technology adoption and diffusion, as well as enabling ongoing technological advancements to meet the industry's evolving needs.

## 5 Conclusion

This study showcased the transformation of food safety management in the California almond industry following *Salmonella* outbreaks linked to raw almonds in 2001 and 2004. These outbreaks brought new attention to microbial contamination risks in almonds and prompted the industry to take corrective action. ABC, an industry commodity board, led the response, coordinating immediate crisis management, efforts to understand the risks and explore *Salmonella*‐control interventions processes, develop best practices, and implement long‐term food safety programs and standards. These actions significantly strengthened the industry's food safety management systems and became a model for successful self‐regulation.

The two primary drivers of improvement identified in this study were the *Salmonella* outbreaks themselves, which acted as catalysts for change and ABC's leadership in driving proactive, coordinated responses. In addition to the management system advancements, the industry has also shown a shift in mentality toward food safety over time. Indicators of change include greater prioritization of food safety, stronger management support and commitment, and decreased resistance to change. External pressures, such as regulatory requirements and third‐party certifications, as well as internal drivers, such as the company‐level commitment and ongoing communication, were influential in shaping this cultural shift. Despite this progress, economic constraints remain an emerging challenge to the long‐term sustainability of food safety investments.

One major outcome of ABC's leadership was the implementation of a mandatory *Salmonella*‐control program. The present study provides a detailed analysis of the California almond industry's remarkable transformation, highlighting how industry‐led initiatives can advance food safety. A conceptual framework integrating the institutional theory and DOI theory is proposed, highlighting the impact of external and internal institutional pressures on decisions about whether to adopt an intervention, while the characteristics of the intervention itself shape decisions about which option to adopt. These findings provide practical insights for other sectors seeking proactive, industry‐driven food safety improvements through collaborative self‐regulation.

## Author Contributions

**Han Chen**: formal analysis, investigation, project administration, writing – original draft, visualization, conceptualization, data curation, software. **Linda J. Harris**: conceptualization, writing – review and editing, supervision, funding acquisition, methodology. **Tim Birmingham**: writing – review and editing, resources, methodology. **Guangwei Huang**: methodology, writing – review and editing, resources. **Yaohua Feng**: funding acquisition, writing – review and editing, conceptualization, methodology, validation.

## Conflicts of Interest

The authors declare no conflicts of interest.

## Supporting information




**Table S1**. Food safety evolution study interview questions
**Table S2**. Numbers meeting minutes and action plan updates available for review in Study 1.
