# Supplementary material for: From Past to Present: Transformation of Food Safety Management and Food Safety Culture in the California Almond Industry
Source: Compr Rev Food Sci Food Saf. 2026 Feb 28;25(2):e70427. doi: 10.1111/1541-4337.70427 (PMC12949663; doi:10.1111/1541-4337.70427)
Supplement: Supplementary file 1 — Table S1. Food safety evolution study interview questions Table S2. Numbers meeting minutes and action plan updates available for review in Study 1. [file CRF3-25-e70427-s001.docx]

Table S1. Food safety evolution study interview questions

| **Question Number** | **Questions** |
| --- | --- |
| **Background** | |
| 1 | (*For experts from industry or government*) What are your current position and responsibilities in your company/organization?  (*For experts from academia)* What are your current position and areas of research?  How many years have you worked in the almond industry in total? |
| 2 | How many years have you worked with almonds in total?   - Which year did you start to work with almonds/enter the almond industry? - Do you have other experiences in the food industry (or work with other food commodities)? |
| **History – For experts who entered industry prior to 2001 (1^st^ outbreak)** | |
| 1 | When you entered this field before 2001, how would you describe the food safety awareness and practices around almonds at the time?   - How does this compare to (*quoting their experience with other food commodities)* industry? |
| 2 | When the first outbreak happened in 2001, how did you feel when this outbreak happened? |
| 3 | Do you remember how the almond industry and government agencies responded to the 2001 outbreak? Were there any different voices? (i.e., changes in food safety measures, and regulations) |
| 4 | What actions did the industry take as a result of this outbreak (i.e., setting up new committees, funding more research, having more meetings….) |
| 5 | How did this outbreak influence your career? (i.e., changes in job/responsibilities) |
| 6 | What were the challenges for the almond industry after this outbreak? |
| 7 | Fast forward to the second outbreak in 2004.  Did you feel the same way (*quoting their description of feeling*) when this second outbreak was announced? |
| 8 | Do you remember how the almond industry and government agencies responded to this outbreak? |
| 9 | You mentioned *(quoting actions mentioned)* in 2001. How did the 2004 outbreak influence those activities? Were there more changes since this outbreak? |
| 10 | How did this second outbreak influence your career? (i.e., changes in job/responsibilities) |
| 11 | What were the challenges for the industry after this outbreak? Are they the same challenges from the 2001 outbreak? |
| 12 | From your perspective, what are the most significant contributing factors that have shaped food safety management in the almond industry over the years? |
| 13 | Is there anything else you want to share about your experiences in food safety in the almond industry in the past years? (e.g., important meeting, and implementation of pasteurization program) |
| **History – For experts who entered industry between 2001 and 2004** | |
| 1 | When you entered this field in (*quoting their year*), how would you describe the food safety awareness and practices around almonds at the time?   - Were there any food safety concerns? If so, what are they? - How does this compare to (*quoting their experience with other food commodities)* industry? |
| 2 | Do you remember how the 2001 almond outbreak was discussed or considered when you first became involved in this field? |
| 3 | How did you feel when the 2004 outbreak happened? |
| 4 | Do you remember how the almond industry and government agencies responded to this outbreak? Were there any different voices? |
| 5 | What actions did the industry take as a result of this outbreak (i.e., setting up new committees, funding more research, having more meetings….) |
| 6 | How did this outbreak influence your career? (i.e., changes in job/responsibilities) |
| 7 | What were the challenges for the almond industry after this outbreak? |
| 8 | From your perspective, what are the most significant contributing factors that have shaped food safety management in the almond industry over the years? |
| 9 | Is there anything else you want to share about your experiences in food safety in the almond industry in the past years? (e.g., important meeting, and implementation of pasteurization program) |
| **History – For experts who entered after 2004 (2^nd^ outbreak)** | |
| 1 | When you entered this field in (*quoting their year*), how would you describe the food safety awareness and practices around almonds at the time?   - Were there any food safety concerns? If so, what are they? - How does this compare to (*quoting their experience with other food commodities)* industry? |
| 2 | How were the 2001 and 2004 almond outbreaks discussed or considered when you first became involved in this field? |
| 3 | What were some challenges for the almond industry since you entered this field?   - Were these challenges being addressed? If so, how? |
| 4 | From your perspective, what are the most significant contributing factors that have shaped food safety management in the almond industry over the years? |
| 5 | Is there anything else you want to share about your experiences in food safety in the almond industry in the past years? (e.g., important meeting, and implementation of pasteurization program) |
| **Present and Future** | |
| 1 | How would you describe the current state of food safety management in the almond industry (e.g., adequate, continuously improving, or with room for improvement)?   - In your opinion, what are some specific areas that the industry is currently handling well? - What are some areas that still need improvement or attention? |
| 2 | Are there any emerging food safety challenges that the industry is currently facing?   - What specific measures do you believe are needed to effectively address these challenges? |
| **Food Safety Culture Evolution** | |
| 1 | Have you heard of the term “food safety culture” before?   - What is your interpretation of the term? (*GFSI definition: shared values, beliefs and norms that affect mindset and behavior toward food safety in, across and throughout an organization*) |
| 2 | On a scale of 0 to 10, how would you describe the food safety culture in the almond industry when you entered the field? (0 is non-existent and 10 represents very high level)   - On the same scale, how would you describe the current food safety culture in the almond industry? - (*If there are differences in rating)* Could you explain why you believe the food safety culture has changed? Can you share specific examples that make you feel the differences? |
| 3 | Are there any challenges you perceive in the continuous improvement of the food safety culture in the almond industry? Why?   - (*If there is any challenge)* How can we help to address those challenges? |

Table S2. Numbers meeting minutes and action plan updates available for review in Study 1.

| **Years** | **Meeting minutes (count)** | **Action plan updates (count)** |
| --- | --- | --- |
| 2002 | 2 | NA ^1^ |
| 2003 | 3 | NA |
| 2004 | 5 | 13 |
| 2005 | 6 | 16 |
| 2006 | 6 | 3 |
| 2007 | 10 | 3 |
| 2008 | 8 | NA |
| 2009 | 7 | NA |
| 2010 | 6 | NA |
| 2011 | 5 | NA |
| 2012 | 7 | NA |
| 2013 | 6 | NA |
| 2014 | 5 | NA |
| 2015 | 6 | NA |
| 2016 | 6 | NA |
| 2017 | 4 | NA |
| 2018 | 5 | NA |
| 2019 | 2 | NA |

^1^ NA, not applicable; Almond Board of California stopped issuing the action plan updates after the implementation of the mandatory *Salmonella-*control program in September 2007.
